# Obesity reduces the anticancer effect of AdipoRon against orthotopic pancreatic cancer in diet-induced obese mice

**DOI:** 10.1038/s41598-021-82617-2

**Published:** 2021-02-03

**Authors:** Keizo Takenaga, Miho Akimoto, Nobuko Koshikawa, Hiroki Nagase

**Affiliations:** 1 Laboratory of Cancer Genetics, Chiba Cancer Center Research Institute, 666-2 Nitona, Chiba, 260-8717 Japan; 2 Department of Biochemistry, Teikyo University School of Medicine, 2-11-1 Kaga, Itabashi-ku, Tokyo, 173-8605 Japan

**Keywords:** Cancer, Cell biology, Molecular medicine, Oncology

## Abstract

The antidiabetic adiponectin receptor agonist AdipoRon has been shown to suppress the tumour growth of human pancreatic cancer cells. Because obesity and diabetes affect pancreatic cancer progression and chemoresistance, we investigated the effect of AdipoRon on orthotopic tumour growth of Panc02 pancreatic cancer cells in DIO (diet-induced obese) prediabetic mice. Administration of AdipoRon into DIO mice fed high-fat diets, in which prediabetic conditions were alleviated to some extent, did not reduce either body weight or tumour growth. However, when the DIO mice were fed low-fat diets, body weight and the blood leptin level gradually decreased, and importantly, AdipoRon became effective in suppressing tumour growth, which was accompanied by increases in necrotic areas and decreases in Ki67-positive cells and tumour microvessels. AdipoRon inhibited cell growth and induced necrotic cell death of Panc02 cells and suppressed angiogenesis of endothelial MSS31 cells. Insulin and IGF-1 only slightly reversed the AdipoRon-induced suppression of Panc02 cell survival but had no effect on the AdipoRon-induced suppression of MSS31 cell angiogenesis. Leptin significantly ameliorated AdipoRon-induced suppression of angiogenesis through inhibition of ERK1/2 activation. These results suggest that obesity-associated factors weaken the anticancer effect of AdipoRon, which indicates the importance of weight loss in combating pancreatic cancer.

## Introduction

Pancreatic cancer is the most lethal cancer. Most of the tumours are already unresectable at diagnosis, and the 5-year survival rate is quite low^1^. It is the twelfth most common cancer in the world and the fourth leading cause of cancer deaths in Japan^1,2^.

Epidemiological studies have suggested that prediabetes and chronic hyperglycaemia and hyperinsulinaemia associated with type 2 diabetes are linked to an increased risk of cancer development and progression^3–6^ and enhance the chemoresistance of various cancers, including pancreatic cancer^7,8^. High glucose accelerates cell proliferation and mesenchymal–epithelial transition of pancreatic cancer cells^9–11^. Furthermore, increased amounts of insulin, insulin-like growth factor-1 (IGF-1) and IGF-binding proteins are detected in the sera and tissues of pancreatic cancer patients^12–14^ and promote tumour growth, metastasis and chemoresistance^12,15–18^. These observations suggest that diabetic conditions could increase the risk of pancreatic cancer and influence diabetes-associated pancreatic cancer growth.

Obesity, which is often associated with type II diabetes, has also been shown to increase pancreatic cancer risk and worsen outcome^19–21^. Furthermore, the level of obesity is proportional to pancreatic cancer growth^22^. Although the underlying mechanisms are still unclear, stimulation of inflammation, elevated levels of insulin, IGF-1, circulating lipids and cytokines and changes in the intestinal microbiome have been proposed^19,20^.

The adipokine leptin is a hormone‑like cytokine secreted by adipose tissues^23^. Leptin blood levels increase in parallel with increases in body weight and fat reserves^24^. Leptin binds to leptin receptors (LepRs), which include six isoforms (LepRa-f). LepRb is the full-length isoform and can activate downstream signal transducer and activator of transcription 3 (STAT3)^23^. Leptin binding to LepR in the hypothalamus regulates the balance between energy and weight by influencing appetite and energy consumption, thereby modulating fat storage and metabolism^23^. Leptin acts as an inflammatory, mitogenic and proangiogenic factor and is therefore linked to cancer cell proliferation, recurrence and tumour angiogenesis. In pancreatic cancer, leptin is highly expressed and increases cancer stem cell phenotypes, proliferation, migration and chemoresistance^23,25–27^.

The adiponectin receptor agonist AdipoRon was discovered as the first orally active antidiabetic drug^27^. Its binding to the adiponectin receptors AdipoR1 and AdipoR2 activates the AMPK, p38MAPK and PPARα pathways and ameliorates impaired glucose tolerance and insulin resistance linked to type 2 diabetes^28^. Recently, we and Messaggio et al. reported that AdipoRon induces cell death in either an AdipoR-independent or AdipoR-dependent manner and suppresses in vivo tumour growth in human pancreatic cancer cells^29,30^. Because obesity and diabetes influence the sensitivity to chemotherapy of pancreatic cancer^26^, we investigated the anticancer efficacy of AdipoRon in high-fat-diet-induced obese (DIO) prediabetic mice orthotopically implanted with mouse Panc02 pancreatic cancer cells. The results showed that obesity suppressed the anticancer effect of AdipoRon.

## Results

### Panc02-Luc-ZsGreen orthotopic tumour growth in DIO mice

**Figure 1:**
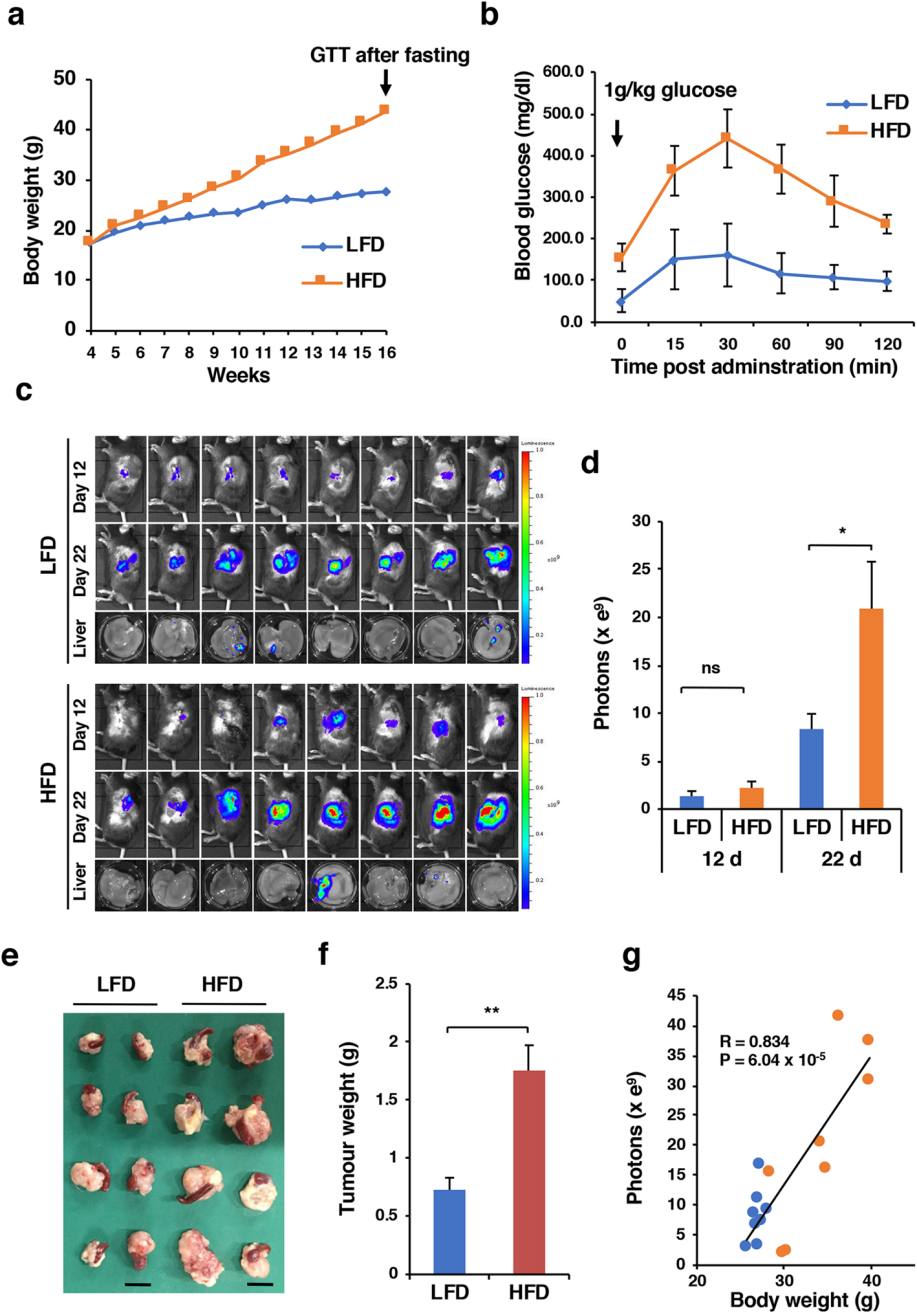
Orthotopic tumour growth of Panc02-Luc-ZsGreen cells in DIO mice. (**A**) Body weight. Four-week-old male C57BL/6J mice were fed either an LFD or an HFD (n = 11 mice for each group). After 16 weeks, a glucose tolerance test (GTT) was performed after overnight fasting. (**B**) GTT. The mice fed an LFD or an HFD (n = 5 mice for each group) were administered 1 g/kg glucose intraperitoneally for the GTT. (**C**) Orthotopic tumour growth and liver metastasis in the mice fed an LFD or an HFD (n = 8 mice for each group). On day 12 and day 22, the mice were subjected to in vivo bioluminescent imaging. (**D**) Tumour growth on day 12 and day 22 represented by photons. Photons from animals’ whole bodies were imaged using the IVIS imaging system. (**E**) Orthotopic tumours with the spleen resected from the mice fed an LFD or an HFD on day 22. Bars 1 cm. (**F**) Tumour weight. (**G**) Correlation between body weight and tumour size (photons). Blue and orange circles represent individual mice in the LFD and HFD groups, respectively. *P < 0.05, **P < 0.01, ns, not significant.

The body weight of mice fed an LFD (control mice) at 16 weeks of age was 27.71 ± 1.81 g (n = 11), and that of those fed an HFD (DIO mice) was 43.69 ± 3.31 g (n = 11) (Fig. 1A). The blood glucose levels of control mice (n = 5) and DIO mice (n = 5) were 52.0 ± 27.6 mg/dl and 155.6 ± 33.2 mg/dl, respectively (P = 4 × 10^–4^). The intraperitoneal glucose tolerance test showed that the clearance of an injected glucose load from the body was slower in DIO mice than in control mice (Fig. 1B), indicating impaired glucose tolerance. We injected Panc02-Luc-ZsGreen cells into the pancreas of these mice and monitored tumour growth. DIO mice developed larger orthotopic tumours than control mice on day 22, as demonstrated by bioluminescent imaging and tumour weight (Fig. 1C–F). The incidence of liver metastasis was not different between control mice (3/8 mice) and DIO mice (2/8 mice) (Fig. 1C). There was a strong positive correlation between body weight and tumour burden (r = 0.834, P < 6.04 × 10^–5^), suggesting that obesity is responsible for the acceleration of Panc02 tumour growth (Fig. 1G).

### Effect of AdipoRon on Panc02-Luc-ZsGreen orthotopic tumour growth

**Figure 2:**
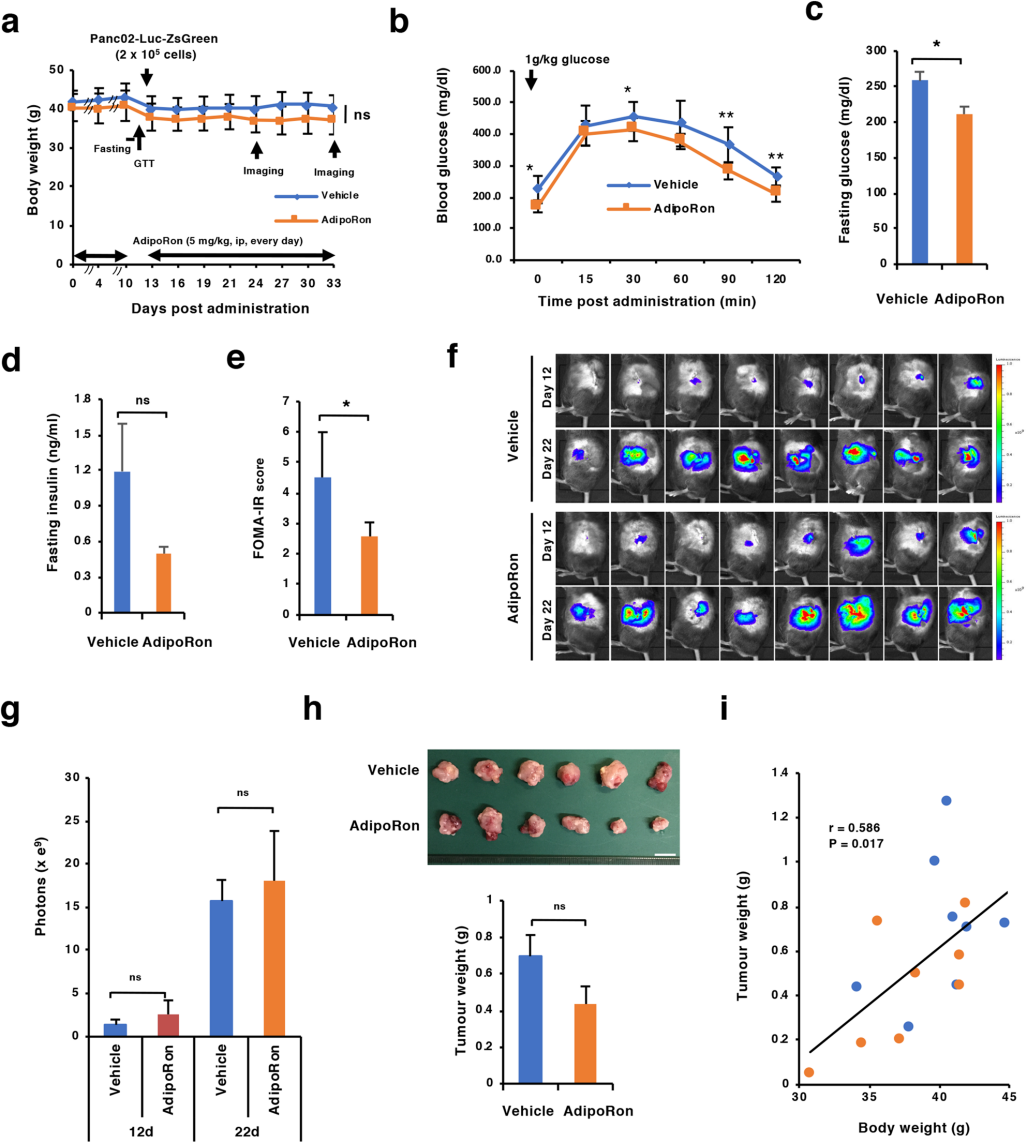
Effect of AdipoRon on orthotopic tumour growth in DIO mice maintained on an HFD. (**A**) Schedule of the experiments and monitoring of body weight. DIO mice were intraperitoneally administered 5 mg/kg AdipoRon or vehicle every day for 10 days, and then the GTT and blood sampling were performed after overnight fasting. On day 11, Panc02-Luc-ZsGreen cells (2 × 10^5^ cells in 50% Matrigel) were injected into the pancreas. From day 13, the mice were intraperitoneally administered 5 mg/kg AdipoRon or vehicle every day until day 30. On day 23 and day 33, in vivo bioluminescent imaging was performed. (**B**) GTT performed on day 11 (n = 5 mice for each group). (**C**) Fasting glucose level (n = 5 mice for each group). Blood was collected on day 11 after fasting. (**D**) Fasting insulin level. Blood was collected on day 11 after fasting (n = 5 mice for each group). (**E**) HOMA-IR score calculated from the fasting glucose level and fasting insulin level in blood. (**F**) In vivo bioluminescent images. Orthotopic tumour growth in the vehicle- and AdipoRon-administered mice (n = 8 mice for each group) was assessed on day 23 (12 days after tumour implantation) and day 22 (22 days after tumour implantation). (**G**) Tumour growth on day 12 and day 22 represented by photons. Photons from animals’ whole bodies were imaged using the IVIS imaging system. (**H**) Tumours and tumour weight. Bar 1 cm. (**I**) Correlation between body weight and tumour weight. Blue and orange circles represent individual mice in the LFD and HFD groups, respectively. *P < 0.05, **P < 0.01, ns, not significant.

We first addressed whether obesity-associated prediabetic conditions influence the suppressive effect of AdipoRon on the orthotopic tumour growth of Panc02-Luc-ZsGreen cells. We chose to administer 5 mg/kg AdipoRon into the mice because the dose is reported to be effective in relieving diabetic symptoms^28^. AdipoRon was intraperitoneally administered once daily into DIO mice for 10 days. Although body weight was not changed (Fig. 2A), reductions in impaired glucose tolerance, fasting insulin level and insulin resistance represented by the HOMA-IR value were indeed observed in the AdipoRon-administered group compared to the solvent-administered group (Fig. 2B–E), confirming the efficacy of AdipoRon as an antidiabetic drug. Then, the DIO mice that had received AdipoRon for 10 days were orthotopically implanted with Panc02-Luc-ZsGreen cells and continuously received daily administration of solvent alone or AdipoRon. The mice were maintained on an HFD. As a result, it appeared that the average body weight of the DIO mice was not significantly reduced by AdipoRon administration over the course of the experiments (Fig. 2A). Moreover, AdipoRon did not significantly affect the growth of orthotopic tumours, as evidenced by bioluminescent imaging and tumour weight (Fig. 2F–H). Notably, there was still a significant positive correlation between body weight and tumour weight at the end of the experiments (Fig. 2I). Thus, administration of 5 mg/kg AdipoRon apparently attenuated the prediabetic conditions to some extent but did not have statistically significant suppressive effects on obesity and tumour growth.

**Figure 3:**
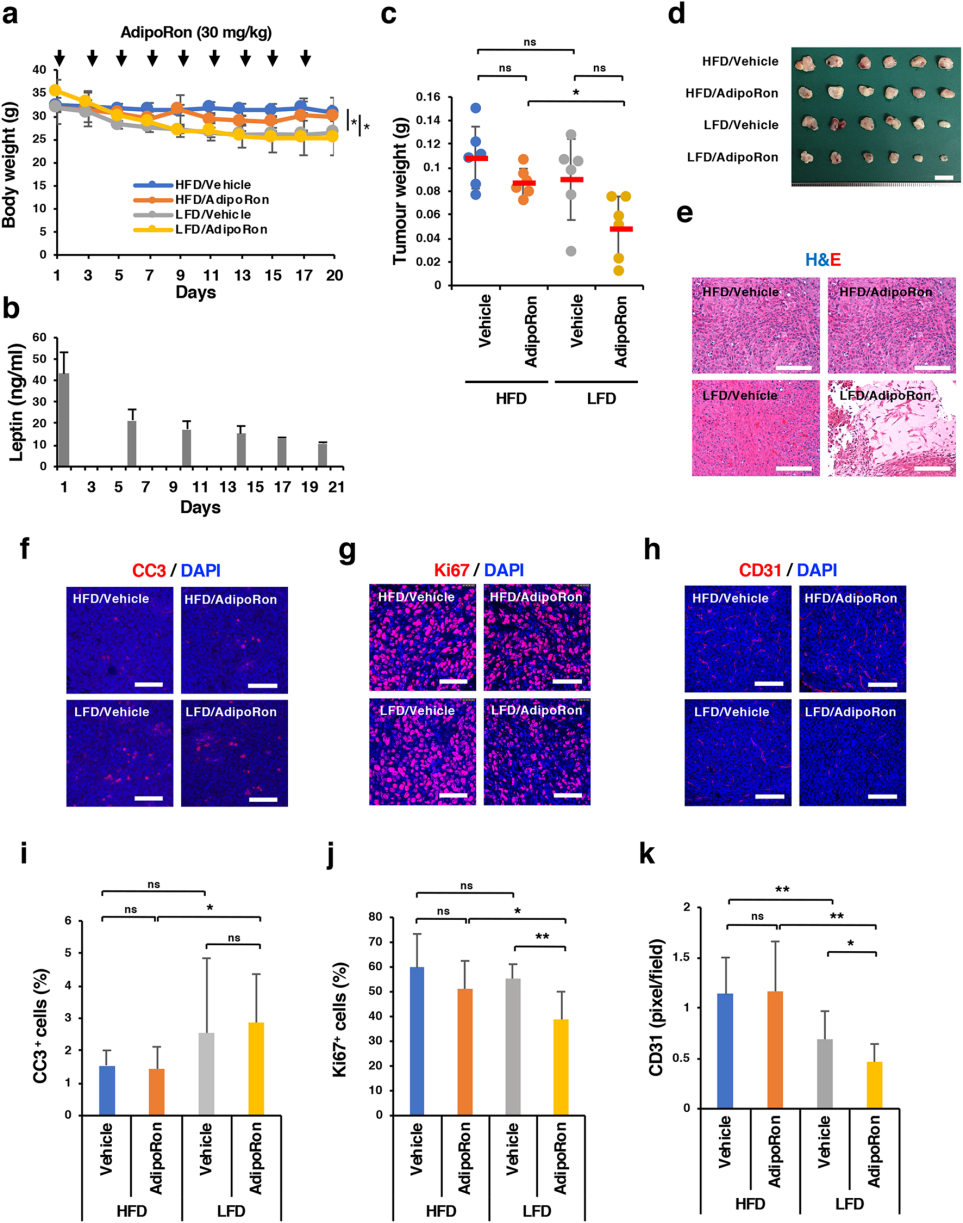
Effect of body weight loss on the anticancer effect of AdipoRon. (**A**) Assessment of body weight after the change of diet from an HFD to an LFD and scheduled AdipoRon administration. DIO mice were orthotopically implanted with Panc02-Luc-ZsGreen cells (2 × 10^5^ cells in 50% Matrigel) on day 0 and then randomly divided into four groups (n = 6 mice for each group); mice fed an HFD and intraperitoneally administered vehicle (HFD/Vehicle group) or 30 mg/kg AdipoRon every other day (HFD/AdipoRon group) and mice fed an LFD and intraperitoneally administered vehicle (LFD/Vehicle group) or 30 mg/kg AdipoRon every other day (LFD/AdipoRon group). (**B**) Serum leptin levels after the change in diet from an HFD to an LFD. The DIO mice (n = 5 mice) were fed an LFD from day 1. (**C**) Orthotopic tumour weight in each group (n = 6 mice). (**D**) Tumours. Bar 1 cm. (**E**) H&E staining of tumour tissues from each group. Bars 200 μm. (**F**) Immunostaining of cleaved caspase-3 (CC3) in tumour tissues of each group. Bars 50 μm. (**G**) Immunostaining of Ki67 in tumour tissues of each group. Bars 50 μm. (**H**) H&E staining of tumour tissues from each group. Bars 50 μm. (**I**) The percentage of CC3-positive cells in tumour tissues of each group. (**J**) The percentage of Ki67-positive cells in tumour tissues of each group. (**K**) The number of tumour vessels in tumour tissues of each group. *P < 0.05, **P < 0.01, ns, not significant.

Next, we divided the DIO mice into four groups after orthotopic implantation of Panc02-Luc-ZsGreen cells: HFD/Vehicle group (n = 6): mice fed an HFD continuously and administered vehicle alone intraperitoneally every other day; HFD/AdipoRon group (n = 6): mice fed an HFD continuously and administered AdipoRon (30 mg/kg) intraperitoneally every other day; LFD/Vehicle group (n = 6): mice fed an LFD instead of an HFD and administered vehicle alone intraperitoneally every other day; and LFD/AdipoRon group (n = 6): mice fed an LFD instead of an HFD and administered AdipoRon (30 mg/kg) intraperitoneally every other day. We confirmed body weight loss and a decrease in serum leptin levels in the LFD groups (Fig. 3A,B). Again, as long as the DIO mice were fed an HFD, we did not observe a significant reduction in body weight or tumour growth by AdipoRon (Fig. 3A,C). However, when the diet was changed from an HFD to an LFD, tumour growth tended to be retarded by AdipoRon, although the differences were not statistically significant (Fig. 3A,C). Importantly, AdipoRon suppressed tumour growth more significantly in the LFD/AdipoRon group than in the HFD/AdipoRon group (Fig. 3C,D). In agreement with these results, haematoxylin and eosin staining demonstrated a noticeable increase in necrotic areas in the tumour tissues of the LFD/AdipoRon group (Fig. 3E). Immunohistochemical analyses showed that although there was a statistically significant difference in the percentage of cleaved caspase-3 (CC3)-positive cells between the LFD/AdipoRon group and the HFD/AdipoRon group, the percentage was too low to explain the difference in the inhibition of tumour growth (Fig. 3F,I). On the other hand, the reduction in the number of Ki67-positive cells (Fig. 3G,J) and decrease in CD31-positive tumour microvessels (Fig. 3H,K) in the LFD/AdipoRon group were more remarkable than those in the HFD/AdipoRon group, suggesting the possibility that suppression of angiogenesis was partially responsible for the growth inhibition and necrosis. These observations suggested that obesity reduced the anticancer effect of AdipoRon, possibly through suppressing the effects of AdipoRon on cell growth and tumour angiogenesis.

### Effect of AdipoRon and obesity-related factors on the survival of Panc02-Luc-ZsGreen cells in vitro

**Figure 4:**
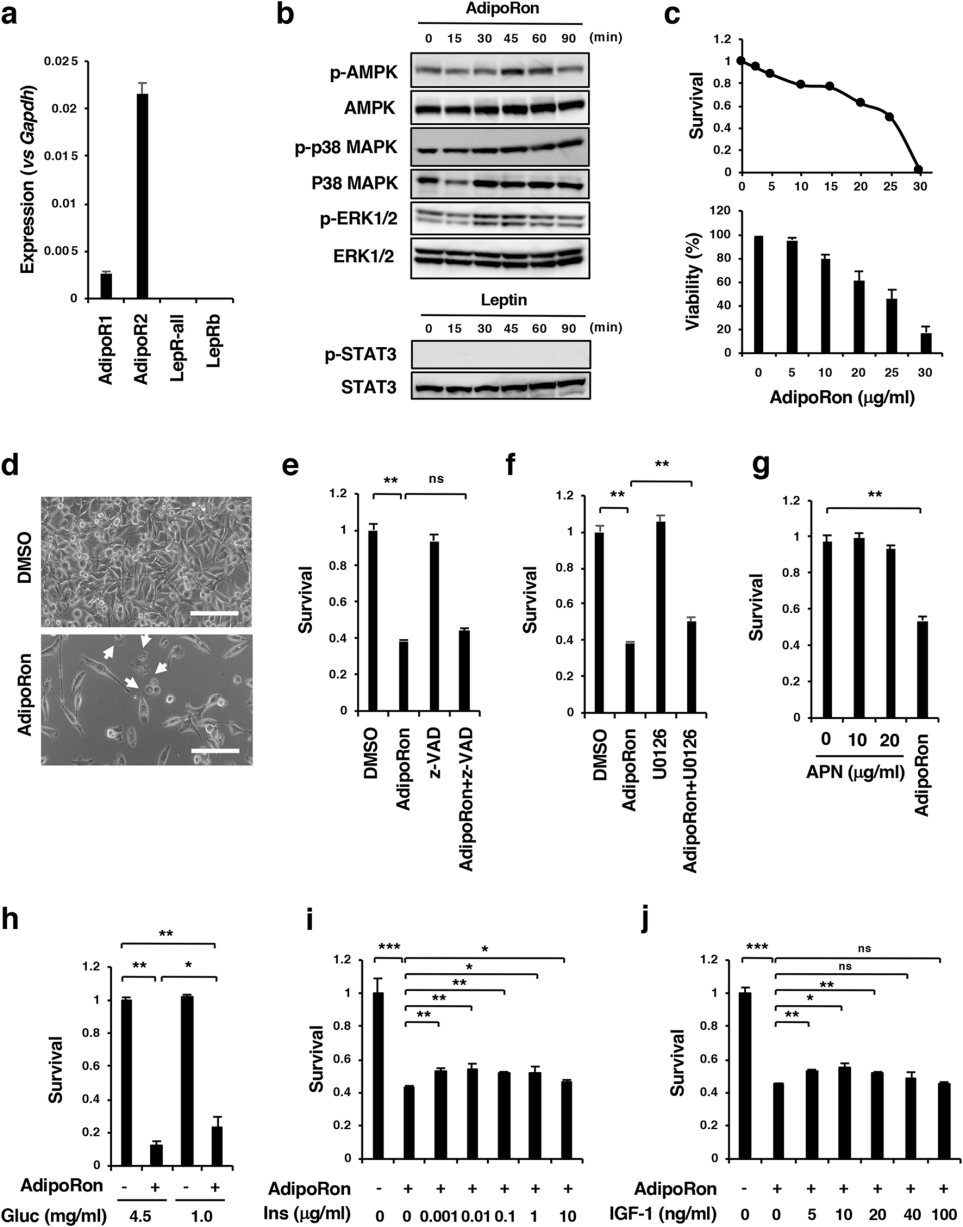
Effects of AdipoRon and obesity-related factors on the survival of Panc02-Luc-ZsGreen cells. (**A**) qRT-PCR analysis of the expression of AdipoRs and LepRs. (**B**) Effects of AdipoRon on the activation of AMPK, p38 MAPK, ERK1/2 and STAT3. The cells were incubated with 25 μg/ml AdipoRon for the indicated times. The uncropped images are shown in Supplementary Fig. S11-1. (**C**) Cell survival. The cells were incubated with various concentrations of AdipoRon for 2 days. The MTT assay and trypan blue dye exclusion test were used for the measurements of cell survival and viability, respectively. The value for each point represents triplicate measurements. (**D**) Cell morphology. The cells were cultured with 30 μg/ml AdipoRon for 2 days. White arrows indicate the cells showing cytoplasmic swelling with large pieces blebbing from the plasma membrane. (**E**) Effect of z-VAD-fmk on AdipoRon-induced cell growth inhibition. The cells were treated with z-VAD-fmk (50 μM) in the presence or absence of AdipoRon (25 μg/ml) for 2 days. (**F**) Effect of U0126 on AdipoRon-induced cell growth inhibition. The cells were treated with U0126 (10 μM) in the presence or absence of AdipoRon (25 μg/ml) for 2 days. (**G**) Effect of adiponectin on cell survival. The cells were incubated with 10 μg/ml or 20 μg/ml adiponectin (APN) and 25 μg/ml AdipoRon for 2 days. (**H**) Effect of glucose on AdipoRon-induced cell growth inhibition. The cells were cultured in low-glucose (1 mg/ml glucose) or high-glucose (4.5 mg/ml) medium containing 10% FBS in the presence or absence of 25 μg/ml AdipoRon. (**I**) Effect of insulin on AdipoRon-induced cell growth inhibition. The cells were incubated with various concentrations of insulin in high-glucose DMEM containing 10% FBS in the presence or absence of 25 μg/ml AdipoRon. (**J**) Effect of IGF-1 on AdipoRon-induced cell growth inhibition. The cells were incubated with various concentrations of IGF-1 in high-glucose DMEM containing 10% FBS in the presence or absence of 25 μg/ml AdipoRon. Cell survival and cell viability were measured with MTT assay and trypan blue staining, respectively. *P < 0.05, **P < 0.01, ns, not significant.

To obtain any clues about the molecular basis for the inhibitory effect of obesity on the antitumour action of AdipoRon, we first examined the effect of AdipoRon and obesity-related factors on the survival of Panc02-Luc-ZsGreen cells. qRT-PCR analyses showed the expression of AdipoR1 and AdipoR2, but not LepRs, in the cells (Fig. 4A). Treatment of the cells with AdipoRon enhanced the phosphorylation of AMPK and p38 MAPK, indicating the functionality of the AdipoRs, whereas as expected, leptin did not increase the phosphorylation of STAT3 (Fig. 4B). AdipoRon caused cell growth inhibition accompanied by cell death in a dose-dependent manner during incubation for 2 days (Fig. 4C,D). The pan-caspase inhibitor z-VAD-fmk did not enhance the survival of AdipoRon-treated cells (Fig. 4E), suggesting that AdipoRon did not substantially induce caspase-dependent apoptosis in the cells. As observed in human pancreatic cancer MIAPaCa-2 cells^29^, AdipoRon induced cytoplasmic swelling with large pieces blebbing from the plasma membrane and eventual cytolysis featuring necrotic cell death (Fig. 4D, Supplementary Fig. S1) and transiently increased the phosphorylation of ERK1/2 (Fig. 4B). The MEK inhibitor U0126 slightly recovered the survival of AdipoRon-treated cells (Fig. 4F). Unlike AdipoRon, adiponectin (APN) did not inhibit survival of the cells (Fig. 4G, Supplementary Fig. S2). These results imply that, similar to the case for MIAPaCa-2 cells^29^, AdipoRon-induced suppression of Panc02-Luc-ZsGreen cell survival is AdipoR-independent. We then examined the effects of several obesity-related factors (glucose, insulin, IGF-1, leptin and palmitic acid) on the growth of Panc02 cells and found that glucose, insulin, IGF-1 or leptin had no direct influence on growth (Supplementary Figs. S3, S4, S5), although among these four factors, we found insulin and IGF-1 to trigger a mild recovery of AdipoRon-induced suppression of cell survival at lower concentrations (Fig. 4H–J, Supplementary Fig. S5), while the administration of palmitic acid led to apparent lipotoxicity and the augmentation of AdipoRon’s suppressive effect on the survival of Panc02-Luc-ZsGreen cells (Supplementary Fig. S6). To examine the possible existence of systemic factors related to the promotion of Panc02-Luc-ZsGreen cell growth and the impairment of AdipoRon’s effect, we sought to examine the difference in the survival of Panc02-Luc-ZsGreen cells in cultures supplemented with 10% serum from mice under either LFD or HFD regimens in the presence or absence of AdipoRon. We observed that HFD serum appeared to have no effect on either cell growth or observable differences in samples treated with AdipoRon (Supplementary Fig. S7), although the sera from HFD-fed mice exhibited elevated ratios of leptin/APN compared to the LFD group (Supplementary Fig. S7). These results, when taken together appeared to indicate that obesity-related factors did not agitate Panc02-Luc-ZsGreen cells and attenuate the effect of AdipoRon in tumour suppression.

### Effect of AdipoRon and obesity-related factors on angiogenesis in vitro

**Figure 5:**
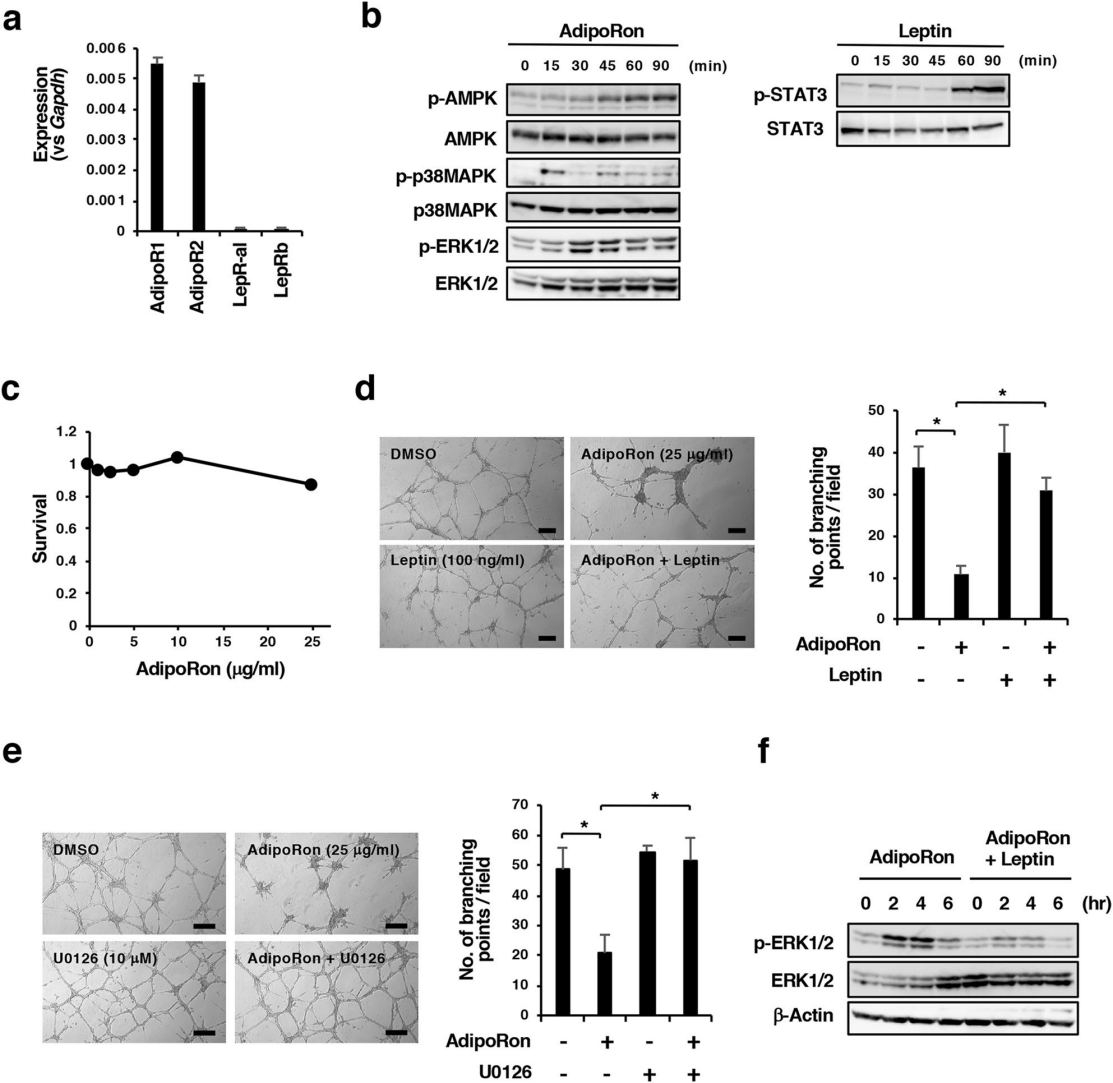
Effects of AdipoRon and leptin on the tube formation of MSS31 cells. (**A**) qRT-PCR analysis of the expression of AdipoRs and LepRs. (**B**) Effects of AdipoRon and leptin on the activation of AMPK, p38 MAPK and STAT3. The cells were incubated with 25 μg/ml AdipoRon or 0.5 μg/ml leptin for the indicated times. The uncropped images are shown in Supplementary Fig. S11-1. (**C**) Cell survival. The cells were incubated with various concentrations of AdipoRon for 2 days. Cell survival was measured with the MTT assay. The value for each point represents triplicate measurements. (**D**) Effects of AdipoRon and leptin on the tube formation of MSS31 cells. The cells were pretreated with leptin (100 ng/ml) for 1 h and then incubated with vehicle (DMSO) alone or 25 μg/ml AdipoRon plus 100 ng/ml leptin on Matrigel for 16 h. The number of branching points per field is also shown. Bars: 100 μm. *P < 0.01. (**E**) Effects of U0126 on AdipoRon-induced suppression of tube formation of MSS31 cells. The cells were pretreated with U0126 (10 μM) for 1 h and then incubated with vehicle (DMSO) alone or 25 μg/ml AdipoRon plus 10 μM U0126 on Matrigel for 16 h. The number of branching points per field is also shown. Bars: 100 μm. *P < 0.01. (**F**) Effects of leptin on AdipoRon-induced ERK1/2 activation in MSS31 cells. The cells were pretreated with leptin (100 ng/ml) for 1 h and then incubated with vehicle (DMSO) alone or 25 μg/ml AdipoRon plus 100 ng/ml leptin for the indicated times. The uncropped images are shown in Supplementary Fig. S11-2.

Based on the suppression of tumour angiogenesis in the LFD/AdipoRon group (Fig. 3H,K) and our previous data that AdipoRon suppressed tumour angiogenesis in a xenograft model of MIAPaCa-2 cells^29^, we examined the effect of AdipoRon on cell growth and tube formation in MSS31 endothelial cells. qRT-PCR analyses showed the expression of AdipoR1 and AdipoR2, and notably LepRb, to a lesser extent, in the cells (Fig. 5A). AdipoRon induced the phosphorylation of AMPK and p38 MAPK, and leptin induced the phosphorylation of STAT3 (Fig. 5B), indicating that both AdipoRs and LepRb elicit signals into the cells. AdipoRon also induced the phosphorylation of ERK1/2 in the cells (Fig. 5B). In contrast to Panc02-Luc-ZsGreen cells, the growth of MSS31 cells was not inhibited by AdipoRon concentrations up to 25 µg/ml (Fig. 5C). A tube formation assay on Matrigel showed that MSS31 cells formed fine tubes in the presence of HGF, and AdipoRon suppressed tube formation (Fig. 5D). Insulin and IGF-1 did not reverse the AdipoRon-induced suppression of tube formation (Supplementary Fig. S8). However, intriguingly, pretreatment of the cells for 1 h with 100 ng/ml leptin followed by treatment with AdipoRon in the presence of 100 ng/ml leptin alleviated the AdipoRon-induced suppression of tube formation (Fig. 5D). Leptin at 20 ng/ml was also effective (Supplementary Fig. S8), although it did not enhance the growth of MSS31 cells in a number of culture conditions (Supplementary Fig. S9). These results suggest that AdipoRon inhibits tube formation and that leptin reverses this effect. To examine the mechanism by which leptin alleviates the suppression of tube formation by AdipoRon, we focused on ERK1/2 activation because we found that the AMPK inhibitor BML-275 and the p38 MAPK inhibitor SB203580 inhibited tube formation, suggesting that both AMPK and p38 MAPK were involved in enhancing MSS31 tube formation, and both inhibitors did not alleviate the suppressive effect of AdipoRon (Supplementary Fig. S10). On the other hand, the MEK inhibitor U0126 reversed AdipoRon-induced suppression of tube formation (Fig. 5E). We then incubated MSS31 cells that had been pretreated with leptin for 1 h with AdipoRon in the presence of leptin for up to 6 h and examined ERK1/2 activation. As a result, we found that AdipoRon-activated ERK1/2 was suppressed by leptin (Fig. 5F). These results suggest that leptin alleviates the suppressive effect of AdipoRon on tube formation in MSS31 cells, possibly by inhibiting AdipoRon-induced ERK1/2 activation.

## Discussion

We demonstrated here that the orthotopic tumour growth of Panc02-Luc-ZsGreen cells was stimulated by obesity, in good agreement with a previous study^22^. We then investigated the anticancer efficacy of AdipoRon in DIO mice implanted with such cells. The results showed that although AdipoRon at the given dose effectively ameliorated the prediabetic condition in terms of reductions in blood glucose and insulin, the treatment failed to suppress tumour growth. This could be due to the rapid tumour growth of Panc02-Luc-ZsGreen cells in the pancreas, which did not give enough time for the tumour growth suppression by AdipoRon to manifest. However, we realized that glucose, insulin and IGF-1 did not affect the growth of Panc02-Luc-ZsGreen cells even at high concentrations, although these have been reported to influence the proliferation of human pancreatic cancer cells^9–18^. Thus, we concluded that amelioration of the prediabetic condition, at least in terms of blood glucose and insulin levels, by a high dose of AdipoRon was unable to suppress orthotopic tumour growth of the cells in the present experimental setting. However, it should be noted that this does not necessarily exclude the involvement of glucose, insulin, IGF-I and other diabetes-related factors, such as circulating lipids and cytokines, and changes in the intestinal microbiome observed in diabetic patients^19,20^ in the growth of human pancreatic cancer cells. Consistent control of these factors by AdipoRon administration might be able to suppress the growth of pancreatic cancer associated with prediabetes and obesity in humans.

A high dose of AdipoRon was also ineffective in inhibiting Panc02 tumour growth in DIO mice as long as they were fed an HFD. However, it significantly suppressed tumour growth when the diet was changed to an LFD, which resulted in the mice losing weight. Histochemical studies indicated an increase in necrotic areas in the tumours of AdipoRon-administered mice fed an LFD. z-VAD-fmk only slightly reversed the AdipoRon-induced growth inhibition, and only a small fraction of tumour cells was positive for CC3 in both the mouse group fed an HFD and the mouse group fed an LFD. These results excluded apoptosis as a main cause of tumour growth inhibition, although caspase-independent apoptosis could not be completely denied. Because AdipoRon inhibited cell proliferation and concomitantly caused necrotic cell death in Panc02 cells in vitro, the increase in necrotic areas could be a direct effect of AdipoRon on Panc02 cells. In addition, because we previously observed the suppression of tumour angiogenesis in human pancreatic cancer tissues of a xenograft mouse model administered AdipoRon^29^, it is also possible that AdipoRon acted on endothelial cells. Impaired tumour angiogenesis should cause a shortage of nutrients and oxygen supply, which brings about necrotic cell death in tumours and a decrease in cell proliferation. Our results showed a significant decrease in the number of microvessels in AdipoRon-administered mice fed an LFD. Coinciding with this, we found that AdipoRon inhibited tube formation of MSS31 cells at a concentration that did not suppress cell proliferation.

To address what kind of obesity-associated factors reduced the anticancer efficacy of AdipoRon, we examined the effect of glucose, insulin, IGF-1 and leptin on the survival of AdipoRon-treated Panc02-Luc-ZsGreen cells in vitro. The results showed that insulin and IGF-1 but not glucose slightly alleviated the suppressive effect of AdipoRon, which indicates that these factors can reduce the effect of AdipoRon in vivo. However, we believe that this is not the main cause because the effect was small, and amelioration of diabetic conditions by AdipoRon marginally affected tumour growth. Leptin neither enhanced the growth of the cells nor interfered with AdipoRon, a finding consistent with the absence LepRb; additionally we excluded palmitic acid as a tumour growth-enhancing factor since it promoted Panc02-Luc-ZsGreen cell death. Of note, as we found sera from HFD-fed mice not to affect the survival of AdipoRon-treated cells, we hypothesized that obesity-related factors may exert their effect through altering the homeostasis of the host rather than cancer cells.

Our earlier^29^ and aforementioned findings then promoted us to investigate the role of tumour angiogenesis and investigated the effect of insulin, IGF-1 and leptin on tube formation of MSS31 cells that expressed both AdipoRs and LepRb, as do HUVECs^31^. Leptin is said to be pro-angiogenic in the context of wound healing^32–34^ and cancer progression^25,32,37^, and serum leptin levels obviously decreased after a change in diet in accordance with previous findings that the leptin blood level increases as body weight increases and is decreased by initial weight loss^24^. This echoed the possibility that leptin affected the antiangiogenic effect of AdipoRon. Indeed, we found that AdipoRon suppressed tube formation of MSS31 cells on Matrigel and leptin alleviated the suppression of tube formation.

The precise mechanisms by which AdipoRon inhibits tube formation and leptin reduces this inhibition remain to be investigated. AdipoRon activated AMPK, p38 MAPK and ERK1/2 in MSS31 cells. We thought that activation of AMPK and p38MAPK enhanced tube formation because activation of AdipoR signalling acts as a survival signal in MIAPaCa-2 cells^29^. In reality, BML-275 and SB203580 inhibited tube formation. On the other hand, U0126 ameliorated AdipoRon-induced suppression of tube formation, indicating that ERK1/2 activation is a cause of the suppression. ERK1/2 activation may overcome AMPK and p38 MAPK activation, leading to the suppression of tube formation. Intriguingly, leptin suppressed AdipoRon-enhanced ERK1/2 activation, in good agreement with the effect of leptin on AdipoRon-induced suppression of tube formation. Leptin has also been demonstrated to activate Notch signalling, PI-3K/Akt signalling and NF-kB signalling^25^. There might be connections between these signalling pathways and AdipoRon signalling pathways. Further studies are necessary to elucidate the mechanisms.

**Figure 6:**
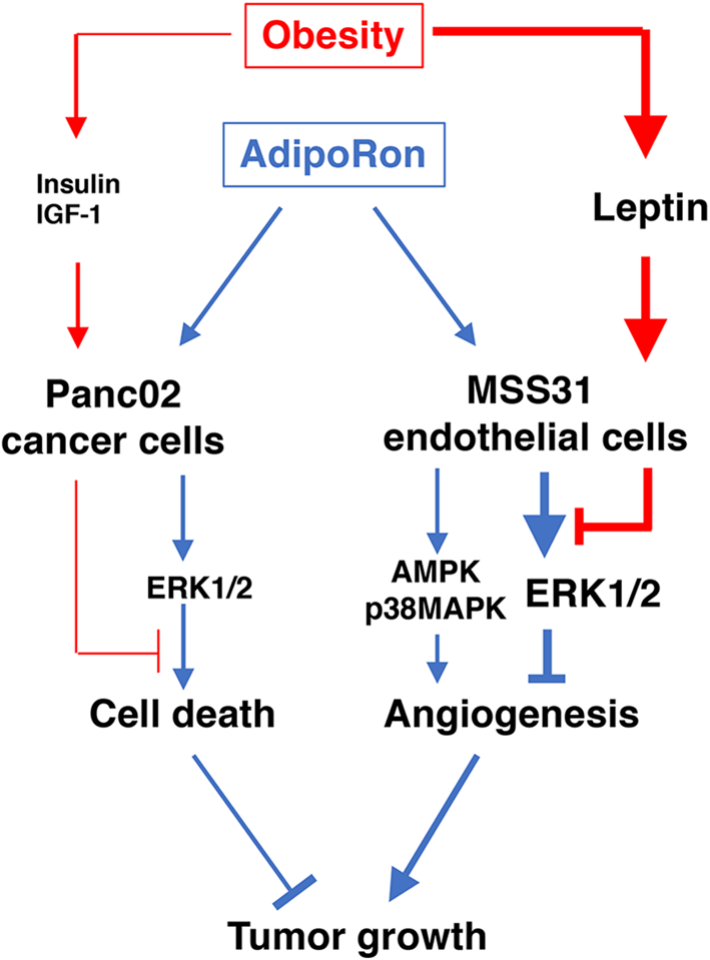
Schematic drawing of the present findings. AdipoRon induces cell death of Panc02 cells partially through ERK1/2 activation. Similarly, AdipoRon suppresses tube formation of MSS31 cells through ERK1/2 activation, which apparently masks the enhancing effect of AMPK and p38 MAPK activation induced by AdipoRon. Obesity-related high insulin and high IGF-1 level slightly recover AdipoRon-induced cell death, while high leptin levels ameliorate the suppression of angiogenesis induced by AdipoRon through inhibition of ERK1/2 activation. Therefore, obesity reduces the anticancer activity of AdipoRon against pancreatic cancer.

In conclusion, AdipoRon suppressed orthotopic Panc02 tumour growth through inhibition of tumour cell survival and tumour angiogenesis in part via ERK1/2 activation, leading to induction of necrotic cell death in tumours, and this effect was ameliorated by obesity-associated factors (Fig. 6). Among these factors, leptin may play an important role in reducing the anticancer activity of AdipoRon. Obesity and leptin signalling have also been reported to enhance chemoresistance to anticancer drugs in human pancreatic cancer^25^. Therefore, a further understanding of why obesity is associated with chemoresistance might lead to novel treatments and enhance the outcome of current therapies against pancreatic cancer.

## Methods

### Cells and cell culture

Mouse pancreatic cancer Panc02-Luc-ZsGreen cells expressing firefly luciferase and ZsGreen were established as described previously^38^. The characteristics of mouse endothelial MSS31 cells were described previously^39–41^. The cells were usually cultured in high-glucose Dulbecco’s modified Eagle’s medium (DMEM) supplemented with 10% foetal bovine serum and 40 μg/ml gentamycin in a humidified atmosphere with 21% O_2_/5% CO_2_.

### Reagent

AdipoRon was purchased from AdipoGen (San Diego, CA, USA). Murine recombinant leptin and murine recombinant adiponectin were obtained from PeproTech (Rocky Hill, NJ, USA). Human recombinant insulin and human recombinant insulin-like growth factor-1 (IGF-1) were supplied by FUJIFILM Wako Pure Chemical Corp. (Osaka, Japan) and Thermo Fisher Scientific (Waltham, MA, USA), respectively. BML-275 was obtained from Santa Cruz Biotechnology (Dallas, TX, USA). SB203580 and U0126 were purchased from Sigma-Aldrich Co. (St. Louis, MO, USA). Palmitic acid and fatty acid-free bovine serum albumin (BSA) were purchased from FUJIFILM Wako. BSA-Palmitic acid conjugate was prepared as described previously^42^.

### qRT-PCR

The expression of AdipoR1, AdipoR2 and LepRs in Panc02-Luc-ZsGreen cells was examined by qRT-PCR. Total RNA was isolated using the RNAeasy Plus Mini Kit (QIAGEN, Hilden, Germany) according to the manufacturer’s protocol. cDNA was synthesized using total RNA and the ReverTra Ace qPCR RT Kit (TOYOBO, Osaka, Japan). Quantitative PCR was performed according to a protocol consisting of an initial denaturation step at 95 °C for 1 min and 40 cycles of denaturation (95 °C for 15 s) and extension (60 °C for 1 min)^29^. The mRNA expression level was normalized to glyceraldehyde-3-phosphate dehydrogenase (*Gapdh*). The primers used were as follows: *AdipoR1* forward primer, 5′-GAGGCTCGAGGTTTCTGAGG-3′; *AdipoR1* reverse primer, 5′-CCCGTCTCACTGCTGGTATG-3′; *AdipoR2* forward primer, 5′-CATAGGGCAGATAGGCTGGC-3′; *AdipoR2* reverse primer, 5′-CTGCAGGTTTGAGACTCCGT-3′; *LepR-all* (LepRa-f) forward primer, 5′-GTGTCAGAAATTCTATGTGGTTTTG-3′; *LepR-all* (LepRa-f) reverse primer, 5′-TGGATATGCCAGGTTAAGTGC-3′; *LepRb* forward primer, 5′-AGTCACAAGATAATGGAGAATAAG-3′; *LepRb* reverse primer, 5′-CTCTACTGGAATGGAACCTT-3′; *Gapdh* forward primer, 5′-TGCACCACCAACTGCTTAG-3′; and *Gapdh* reverse primer, 5′-GGATGCAGGGATGATGTTC-3′.

### Ethics

All animal experiments were performed in compliance with the institutional guidelines for the care and use of animal research and the ARRIVE guidelines. The protocol was approved by the Committee on the Ethics of Animal Experiments of Chiba Cancer Center (Permission Number: 18-1) and the IZUMO Campus Animal Care and Use Committee of Shimane University (Permission Number: IZ26-7).

### Dietary intervention

All mice were housed in the animal centre under specific pathogen-free conditions at a controlled temperature of 23 ± 2 °C and relative humidity of 55 ± 10% and with 12 h light/12 h dark cycles. Four-week-old male C57BL/6J mice (CLEA Japan, Shizuoka, Japan) were randomized to low-fat diet (LFD) and high-fat diet (HFD) groups. Mice in the LFD group and those in the HFD group were fed low-fat diets (LFDs; 20% protein, 70% carbohydrate, and 10% fat) (D12450J) and high-fat diets (HFDs; 20% protein, 20% carbohydrate, and 60% fat) (D12492, Research Diets, Inc., New Brunswick, NJ, USA) ad libitum, respectively.

### Intraperitoneal glucose tolerance test

Overnight (16 h)-fasted mice received an intraperitoneal injection of d-(+)-glucose (1.0 g/kg body weight). Blood samples were collected from the tail vein at 15, 30, 60, 90, and 120 min after glucose injection. Glucose concentrations were measured with a LAB Gluco glucose test meter (ForaCare Japan, Tokyo, Japan).

### Assessment of insulin resistance (HOMA-IR)

Peripheral blood was collected from the tail veins of overnight-fasted mice. After centrifugation of the blood at 1000×*g* for 15 min at 4 °C, the plasma fraction collected from the supernatant was used to estimate blood insulin levels with an Ultra Sensitive Mouse Insulin ELISA Kit (Morinaga Institute of Biological Science, Inc., Yokohama, Japan). The HOMA-IR value was calculated based on the blood glucose concentration and insulin levels^43^.

### Orthotopic tumour injection

For orthotopic implantation, Panc02-Luc-ZsGreen cells (2 × 10^5^ cells) were implanted with 50% Matrigel into the pancreas of anaesthetized mice with medetomidine (0.3 mg/kg)/midazolam (4.0 mg/kg)/butorphanol (5.0 mg/kg). The pancreas together with the spleen was exteriorized through a laparotomy, and the cells were injected using a 30-gauge needle attached to an insulin syringe. The pancreas and spleen were returned to the peritoneal cavity, and the incision was closed with surgical staples. The mice and surgical wounds were observed and evaluated once a day until the mice returned to normal behaviour. The staples were removed 10 days after surgery. The mice were further examined daily for their health, including checks of infection, wound dehiscence and excessive weight loss, and, if necessary, received subcutaneous administration of analgesics to minimize pain and distress. Mice were continued on their diet regimen while tumour growth was monitored. Mice were normally euthanized by CO_2_ inhalation at the end of a study.

### AdipoRon administration

AdipoRon was suspended in 5% DMSO/30% PEG 300/5% Tween 80/PBS. Mice were intraperitoneally administered 5 mg/kg AdipoRon with single daily dosing for 10 days before the glucose tolerance test. Except for the day of Panc02 cell implantation, administration of the same dose of AdipoRon was continued until the end of the experiments. In other experiments, mice were intraperitoneally administered 30 mg/kg AdipoRon every other day after Panc02 cell implantation.

### Bioluminescent imaging

In vivo bioluminescent imaging was performed using the IVIS imaging system (Summit Pharmaceuticals International Corp., Tokyo, Japan). All mice were injected intraperitoneally with 150 mg/kg D-luciferin (PerkinElmer, Waltham, MA, USA) and anaesthetized with 2.5% isoflurane. Ten minutes later, photons from animals’ whole bodies were imaged using the IVIS imaging system according to the manufacturer's instructions. Data were analysed by living image 2.50 software.

### Measurement of serum leptin and adiponectin

Serum leptin and adiponectin levels were measured with a Morinaga Mouse/Rat Leptin ELISA Kit (MIoBS, Yokohama, Japan) and Adiponectin (HMW and Total) ELISA, Mouse Kit (ALPCO Diagnostics, Salem, NH, USA), respectively, according to the manufacturer’s instructions.

### Measurement of cell growth and viability

The MTT (3-(4,5-dimethylthiazol-2-yl)-2,5-diphenyltetrazolium bromide) assay was employed to measure cell growth and viability. Briefly, cells (2 × 10^4^ cells/well for Panc02-Luc-ZsGreen cells and 1 × 10^4^ cells/well for MSS31 endothelial cells) were cultured in 96-well tissue culture plates and treated in triplicate with different concentrations of AdipoRon or solvent (DMSO) alone for 2 days. At the end of the incubation, 10 μl of MTT (2.5 mg/ml) (Sigma-Aldrich, St. Louis, MO, USA) was added to the wells to allow the formation of MTT formazan crystals for 4 h. After the medium was removed, the crystals were solubilized in 100 μl of DMSO. Absorbance was recorded at 590 nm. Cell viability was measured by trypan blue staining.

### Western blot analysis

Panc02-Luc-ZsGreen cells were lysed in RIPA buffer containing Complete Protease Inhibitor Cocktail (Merck, Kenilworth, NJ, USA) and PhosSTOP (Merck). The lysates were centrifuged at 10,000×*g* for 10 min at 4 °C, and the supernatant was used for immunoblot analysis. Proteins were separated by 10% SDS–PAGE under reducing conditions and transferred to an Immobilon-P transfer membrane (Merck Millipore, Billerica, MA, USA). The membrane was incubated with rabbit polyclonal anti-p44/42 MAPK (ERK1/2) (CST, Danvers, MA, USA), rabbit polyclonal anti-phospho-p44/42 MAPK (Thr202/Tyr204) (CST), rabbit polyclonal anti-phospho-AMPKα (Thr172) (CST), rabbit polyclonal anti-AMPKα (CST), rabbit monoclonal anti-phospho-p38 MAPK (Thr180/Tyr182), rabbit monoclonal anti-p38 MAPK (CST), rabbit polyclonal anti-phospho-STAT3 (Tyr705), or rabbit polyclonal anti-STAT3 (CST) antibodies. All primary antibodies were used at 1:1000 dilutions. The membrane was washed extensively with TBS-T and then incubated with HRP-conjugated goat anti-rabbit IgG (1:3000 dilution). ECL Plus Western Blotting Detection Reagent (Amersham Biosciences, Piscataway, NJ, USA) was used for immunodetection.

### Immunohistochemistry

Tumour tissues were fixed in 4% paraformaldehyde solution. The tissues were embedded in paraffin and cut into 2- or 6-µm sections. After heat-induced antigen retrieval in REAL Target Retrieval Solution (DAKO) and subsequent blocking of nonspecific sites with 0.1% normal goat serum/1% BSA, the tissues were immunostained with polyclonal anti-Ki67 antibody (Novus Biologicals, Littleton, CO, USA) and polyclonal anti-cleaved caspase-3 (Asp175) antibody (CST) diluted 1:200 followed by Alexa Fluor 594-conjugated goat anti-rat IgG staining. Sections were counterstained with DAPI and observed under a confocal laser scanning microscope (TCS SC8, Leica Microsystems, Wetzlar, Germany).

### Tube formation assay

MSS31 cells were inoculated onto 12-well Matrigel plates (Geltrex: Reduced Growth Factor Basement Membrane Matrix, 270 μl/well, Life Technologies, Carlsbad, CA) at a cell density of 7 × 10^4^ cells/well. Capillary formation was assessed 16 h after Matrigel culture^32–34^.

### Statistics

Data were presented as mean ± standard deviation. Statistical significance was tested using one-way ANOVA and Tukey’s test. Statistical significance in the difference in tumour weights between two groups was tested by the Mann–Whitney U test. P < 0.05 was considered significant.

## Supplementary Information

Supplementary Information

## Data Availability

All relevant data are within the manuscript and its Supporting Information files.
