# Supplementary material for: Obesity reduces the anticancer effect of AdipoRon against orthotopic pancreatic cancer in diet-induced obese mice
Source: Sci Rep. 2021 Feb 3;11:2923. doi: 10.1038/s41598-021-82617-2 (PMC7859201; doi:10.1038/s41598-021-82617-2)
Supplement: Supplementary file 1 — Supplementary Information [file 41598_2021_82617_MOESM1_ESM.pdf]

Supplementary Information

Obesity reduces the anticancer effect of AdipoRon against orthotopic pancreatic cancer in diet-induced obese mice

Keizo Takenaga, Miho Akimoto, Nobuko Koshikawa, Hiroki Nagase

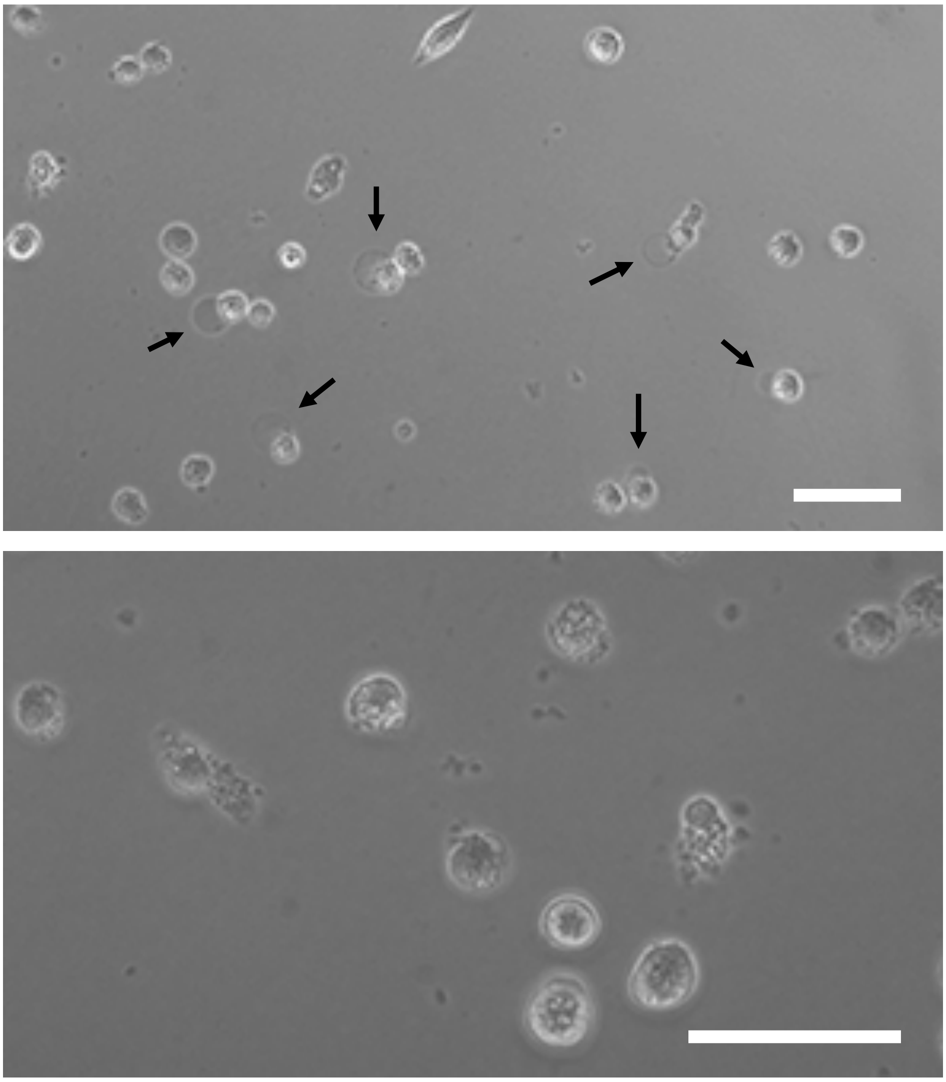

Supplementary Fig. S1. Effects of AdipoRon on the cell death of Panc02-Luc-ZsGreen cells. The cells were treated with 40 μg/ml AdipoRon for 1 day. Arrows indicate the cells showing cytoplasmic swelling with large pieces blebbing from the plasma membrane. Bars: 100 μm.

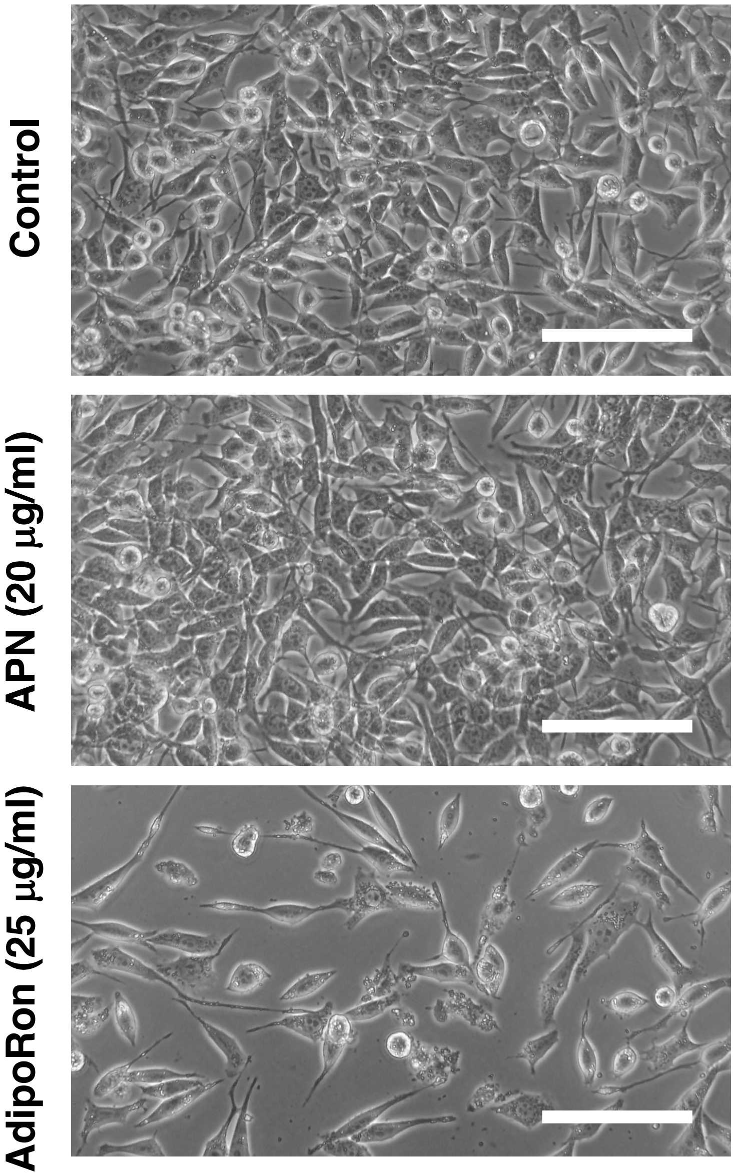

Supplementary Fig. S2. Effects of adiponectin (APN) and AdipoRon on the survival of Panc02-Luc-ZsGreen cells. The cells were treated with vehicle, 20 μg/ml APN or 25 μg/ml AdipoRon for 2 days. Bars: 100 μm.

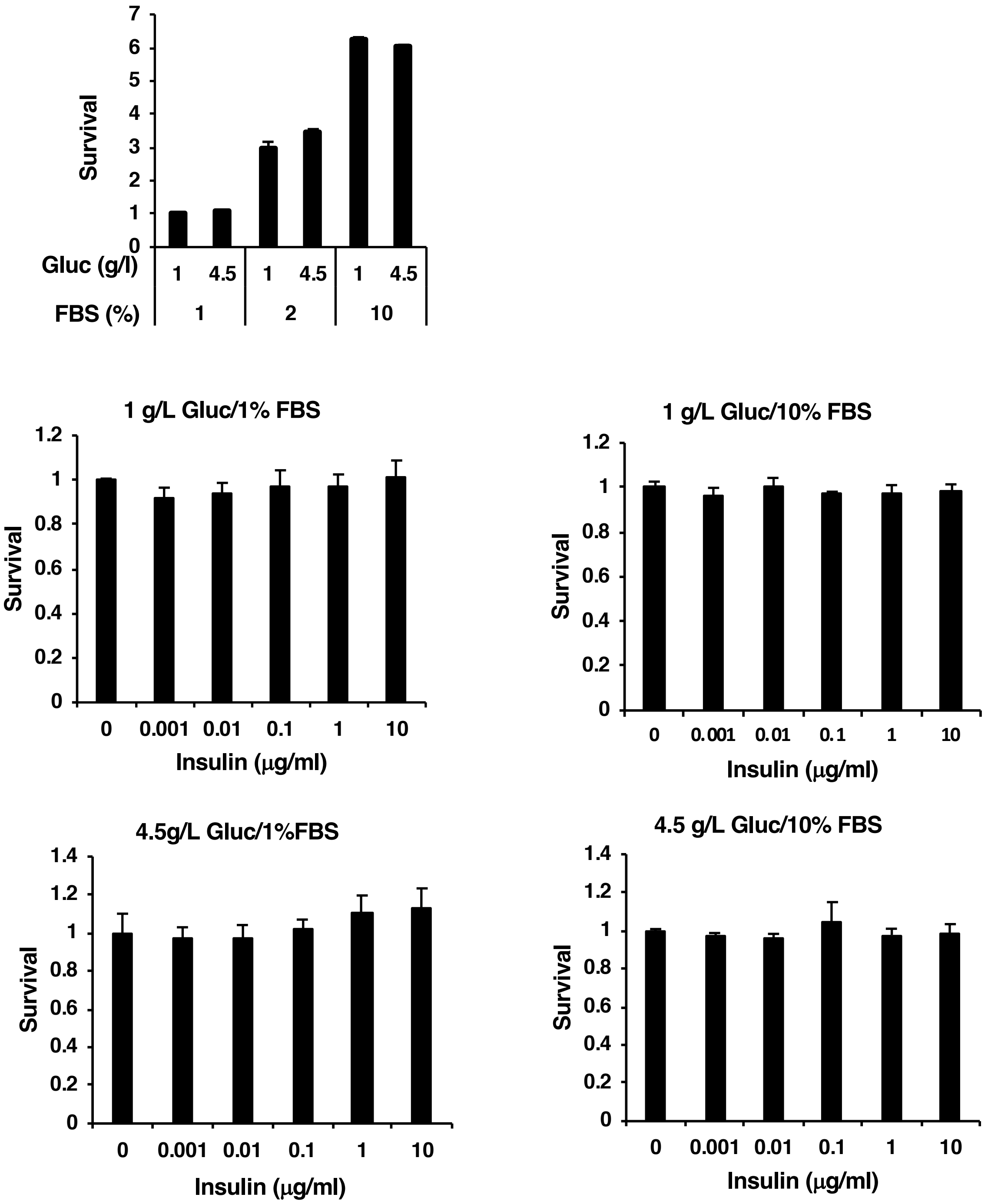

Supplementary Fig. S3. Effects of glucose and insulin on the growth of Panc02-Luc-ZsGreen cells. The cells were cultured with various concentrations of insulin in DMEM containing different concentrations of glucose and FBS for 2 days. Cell growth was assessed with the MTT assay. Bars: SD.

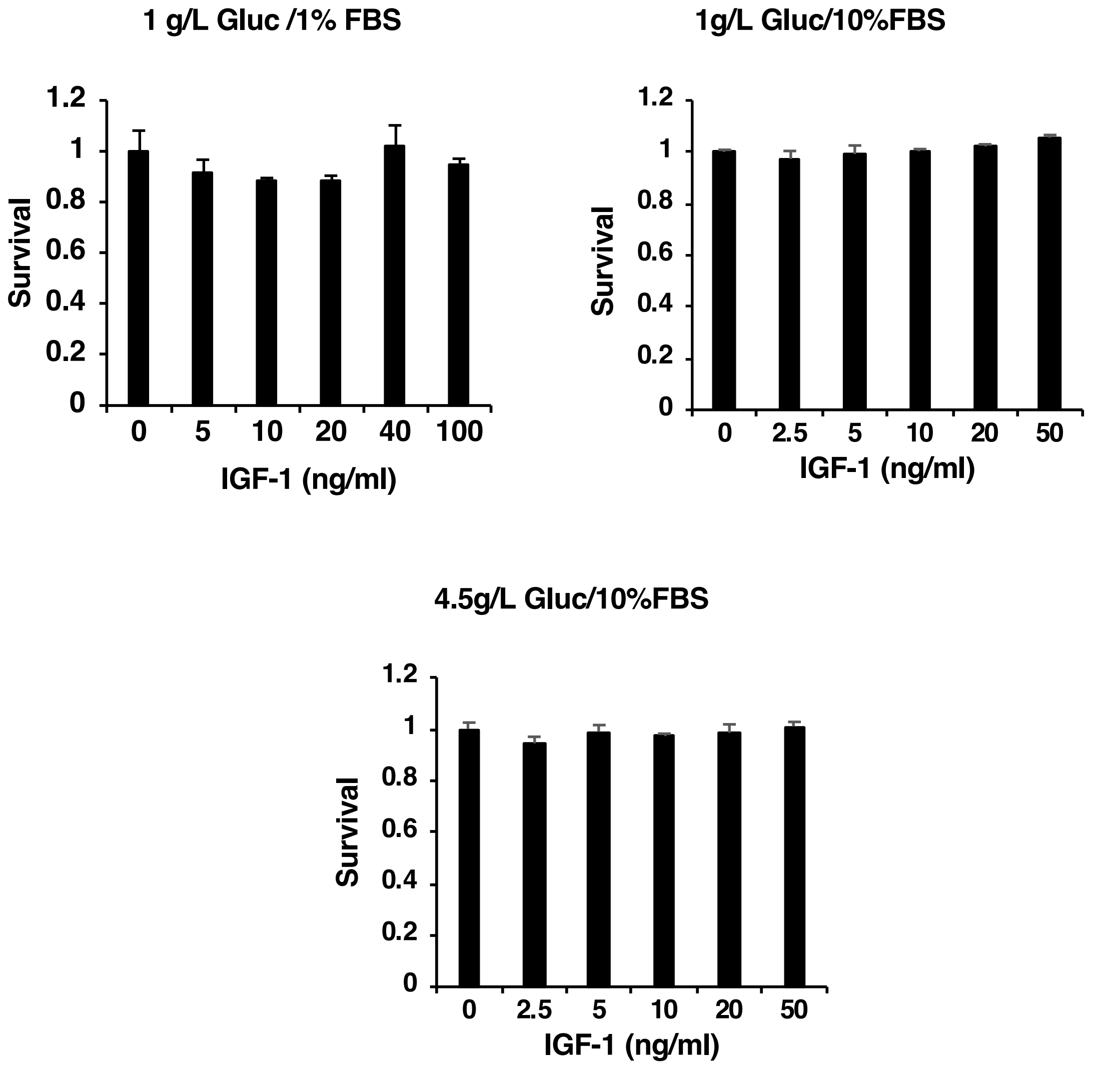

Supplementary Fig. S4. Effects of IGF-1 on the growth of Panc02-Luc-ZsGreen cells. The cells were cultured with various concentrations of IGF-1 in DMEM containing different concentrations of glucose and FBS for 2 days. Cell growth was assessed with the MTT assay. Bars: SD.

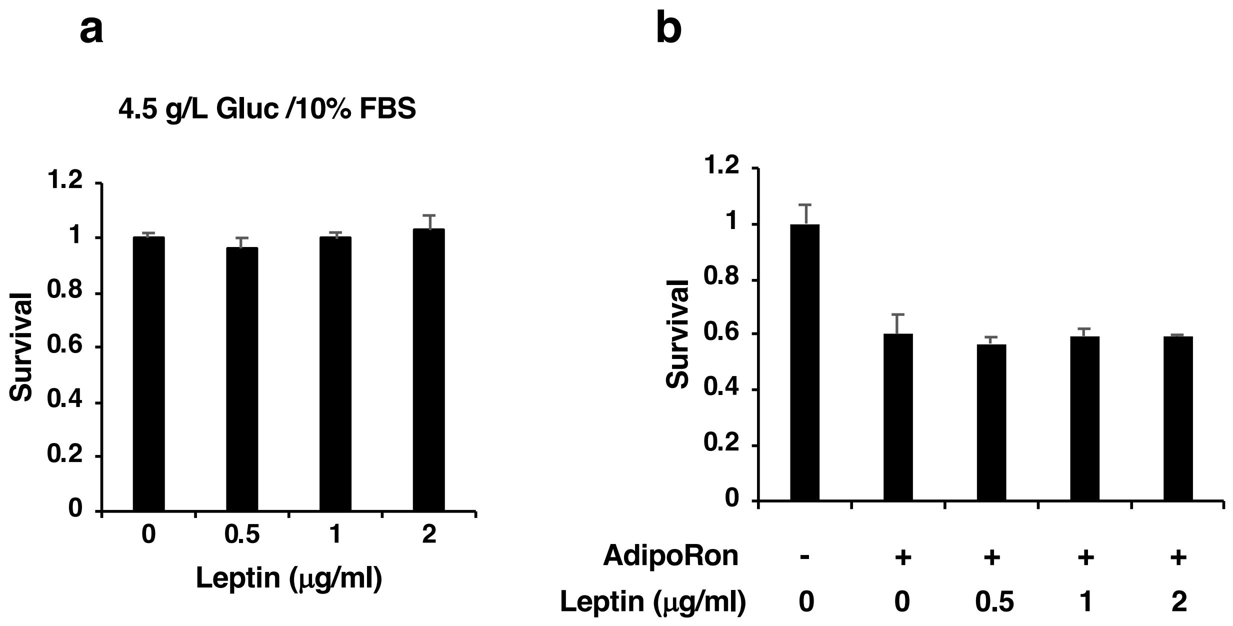

Supplementary Fig. S5. Effects of leptin on the growth of Panc02-Luc-ZsGreen cells and on AdipoRon-induced cell growth inhibition. (A) The cells were cultured with various concentrations of leptin for 2 days. (B) The cells were cultured with various concentrations of leptin in the presence or absence of 25 μg/ml AdipoRon. Cell growth was assessed with the MTT assay. Bars: SD.

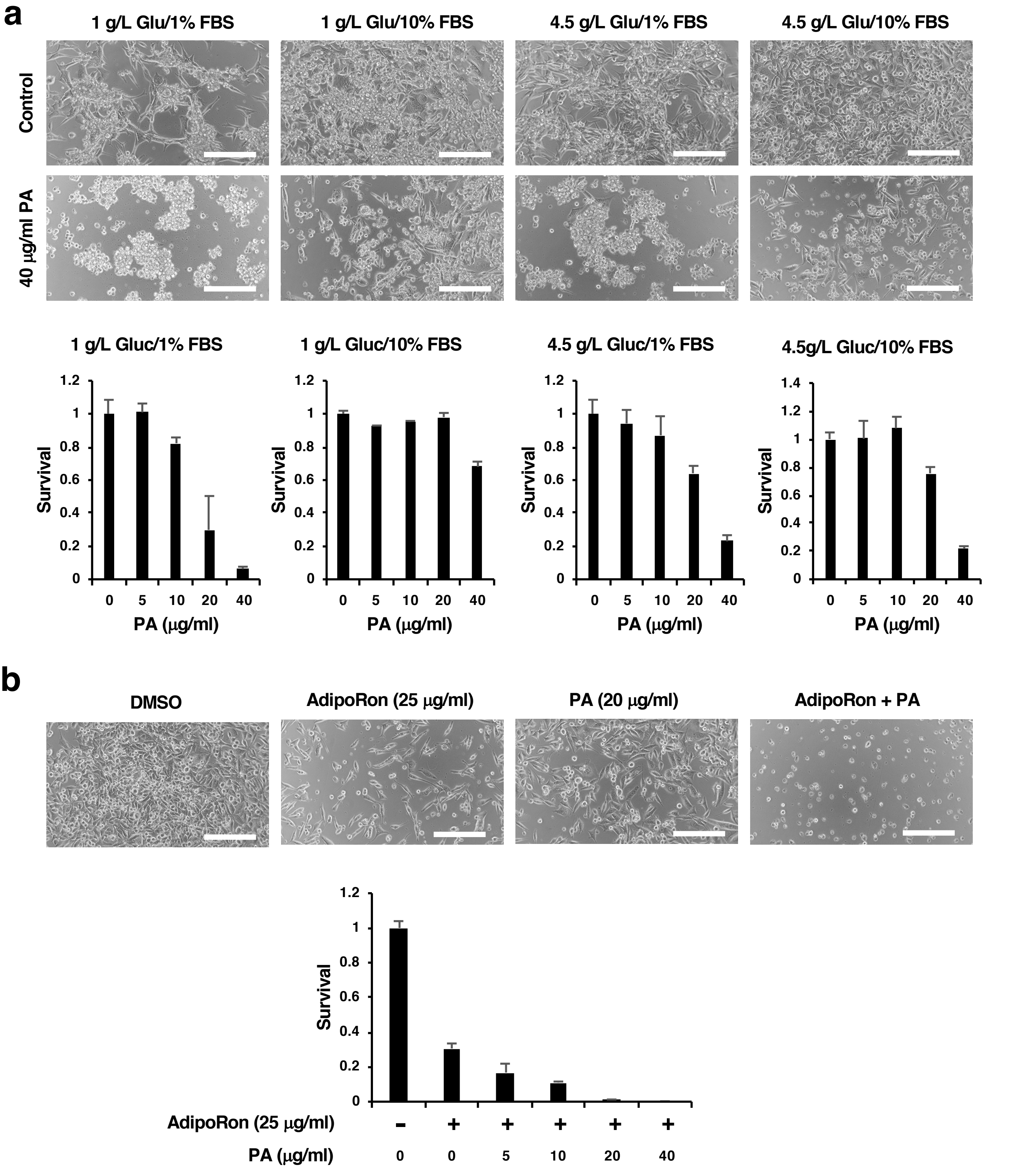

Supplementary Fig. S6. (A) Effects of palmitic acid on the growth of Panc02-Luc-ZsGreen cells. The cells were cultured with various concentrations of palmitic acid (PA) in DMEM containing different concentrations of glucose and FBS for 2 days. Upper panel: Cell morphology of the cells treated with 40 μg/ml PA. Bars: 200 μm. Bottom panel: Cell growth assessed with the MTT assay. (B) Effect of PA on AdipoRon-induced cell growth inhibition. The cells were treated with various concentrations of PA in the presence or absence of 25 μg/ml AdipoRon for 2 days. Upper panel: Cell morphology of the cells treated with AdipoRon in the presence or absence of 20 μg/ml PA. Bars: 200 μm. Bottom panel: Cell growth assessed with the MTT assay. Bars: SD.

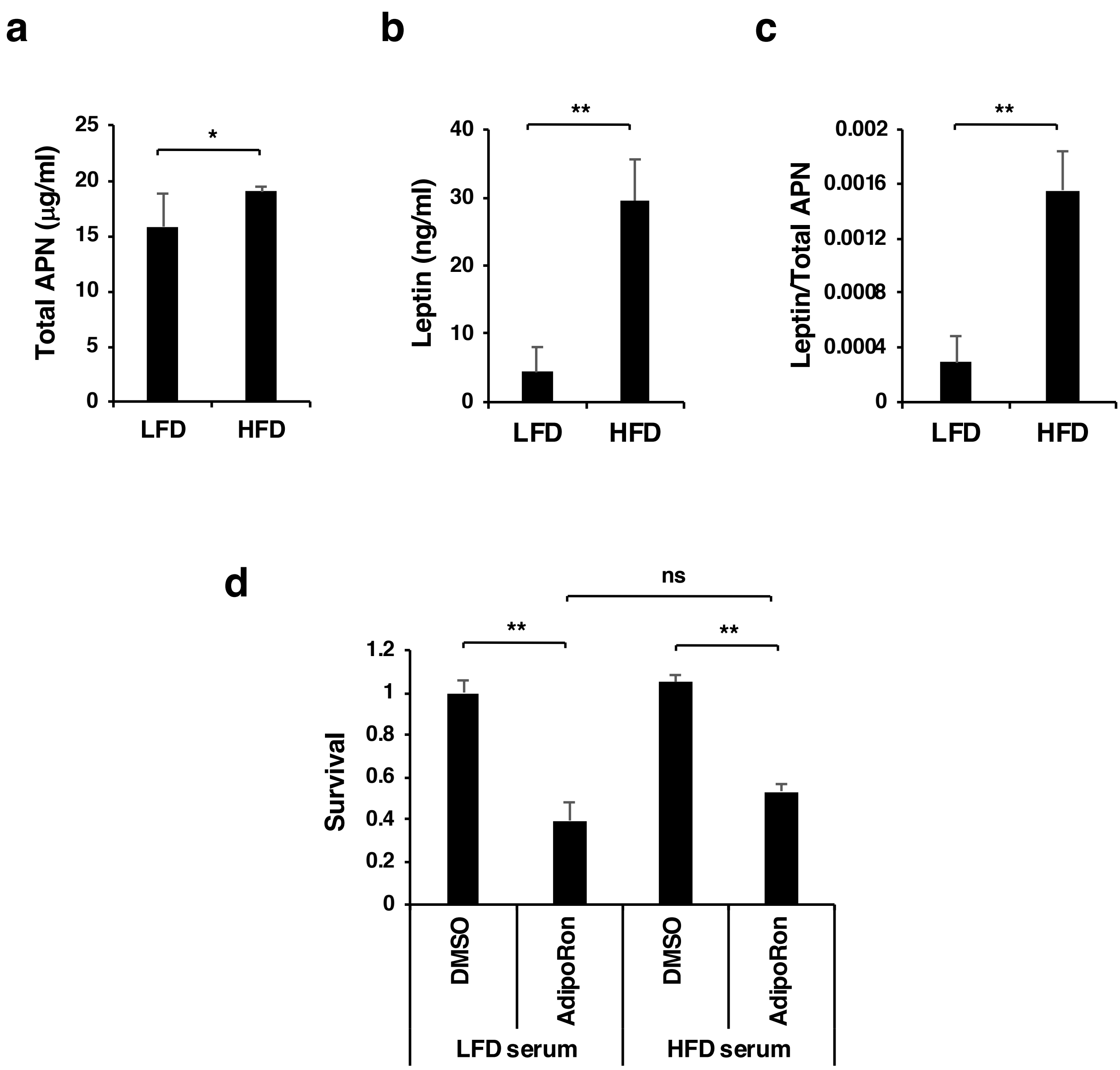

Supplementary Fig. S7. (A) Serum APN level in LFD-fed (n=3) and HFD-fed mice (n=4). (B) Serum leptin level in LFD-fed (n=3) and HFD-fed mice (n=4). (C) Leptin/APN ratio. (D) Effect of AdipoRon on the survival of Panc02-Luc-ZsGreen cells cultured in DMEM medium containing LFD-fed mouse serum or HFD-fed mouse serum. The cells were treated with DMSO or 25 µg/ml AdipoRon in the medium containing 10% serum for 2 days. Cell growth was assessed with the MTT assay. Bars: SD. *P<0.03. **P<0.01. ns, not significant.

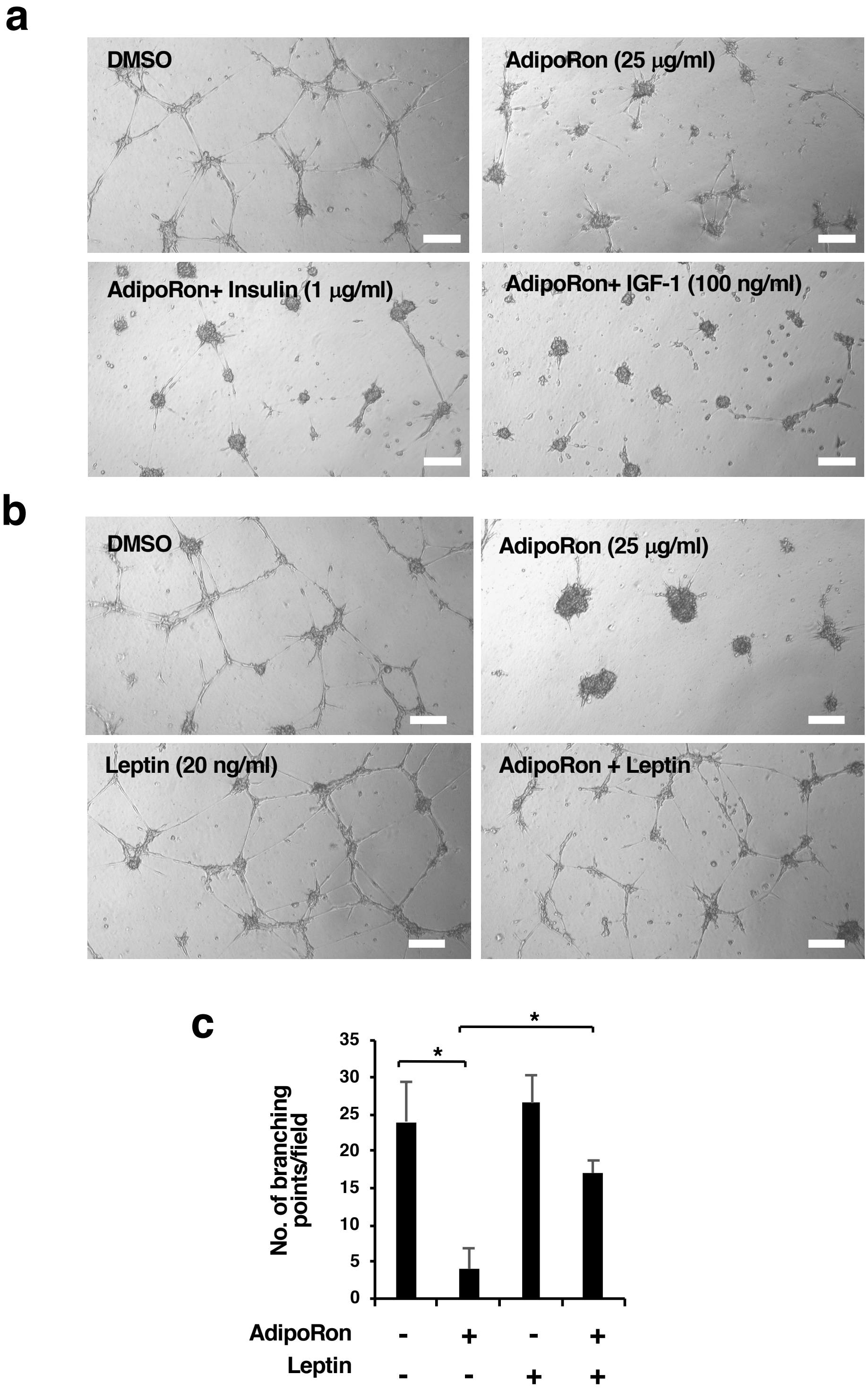

Supplementary Fig. S8. Effects of insulin, IGF-1 and leptin on tube formation of MSS31 cells. A tube formation assay was performed on Matrigel for 16 h in the presence of 50 ng/ml HGF. (a) The cells were pretreated with 1 µg/ml insulin or 100 ng/ml IGF-1 for 1 h and then incubated with insulin or IGF-1 in the presence of DMSO or 25 µg/ml AdipoRon. (b) The cells were pretreated with 20 ng/ml leptin for 1 h and then incubated with leptin in the presence of DMSO or 25 µg/ml AdipoRon. Bars: 100 µm. (c) The number of branching points per field. Bars: SD.

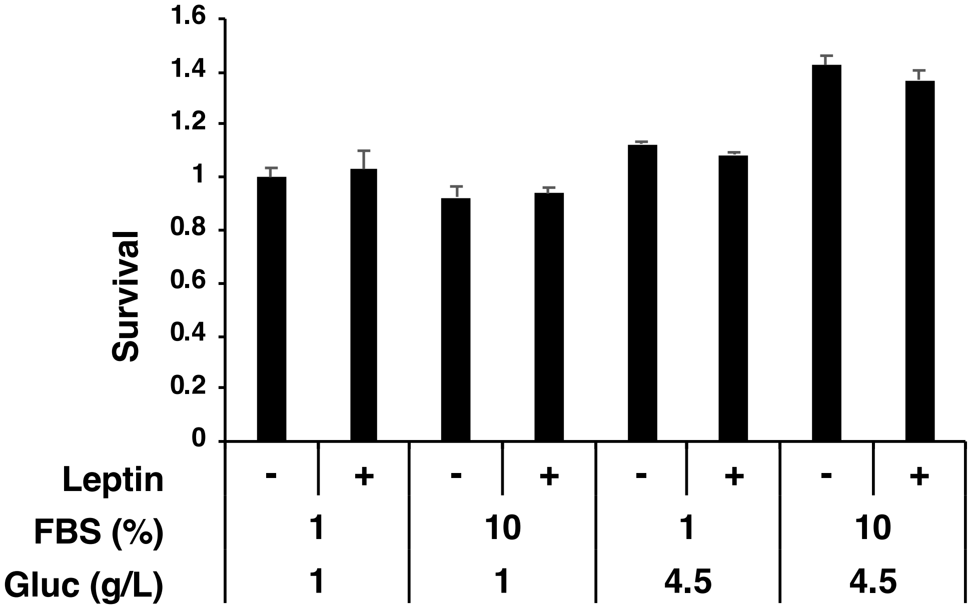

Supplementary Fig. S9. The cells were cultured in DMEM containing different concentrations of glucose and FBS in the presence or absence of leptin (100 ng/ml) for 2 days. Cell growth was assessed with the MTT assay. Bars: SD.

a

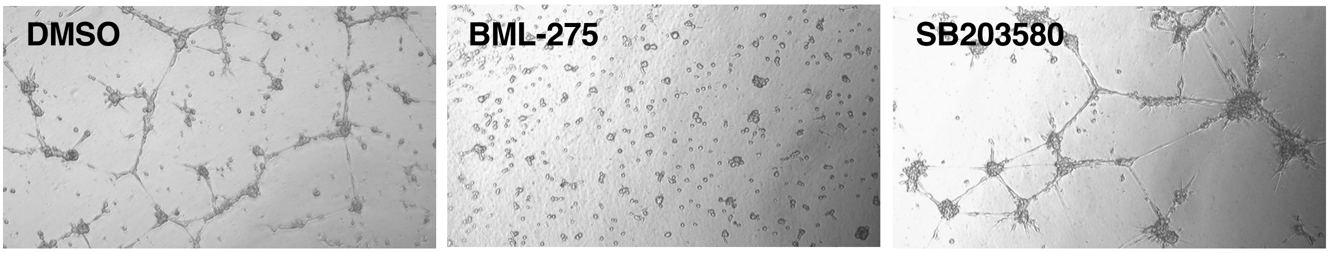

b

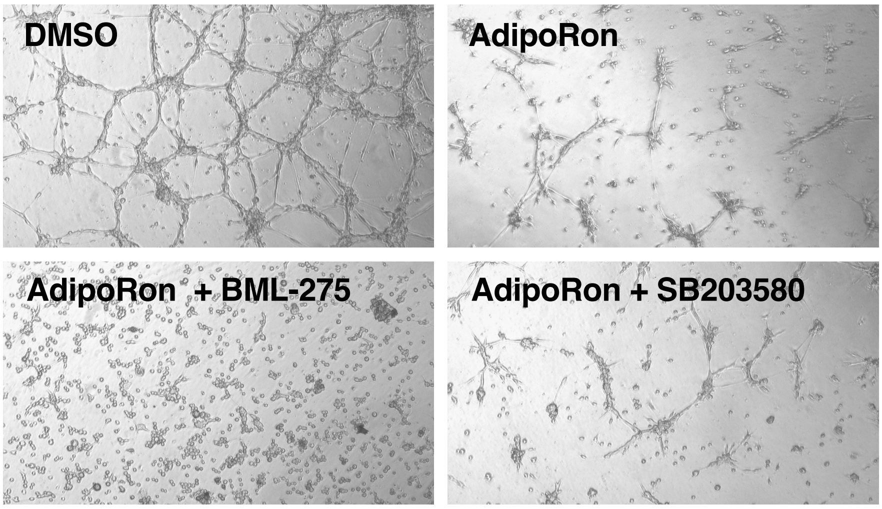

Supplementary Fig. S10. Effects of BML-275 and SB203580 on the tube formation of MSS31 cells. A tube formation assay was performed on Matrigel for 16 h in the presence of 50 ng/ml HGF. (a) Effect of BML-275 and SB203580 on tube formation. (b) Effect of BML-275 and SB203580 on AdipoRon-induced suppression of tube formation.

Fig. 4B

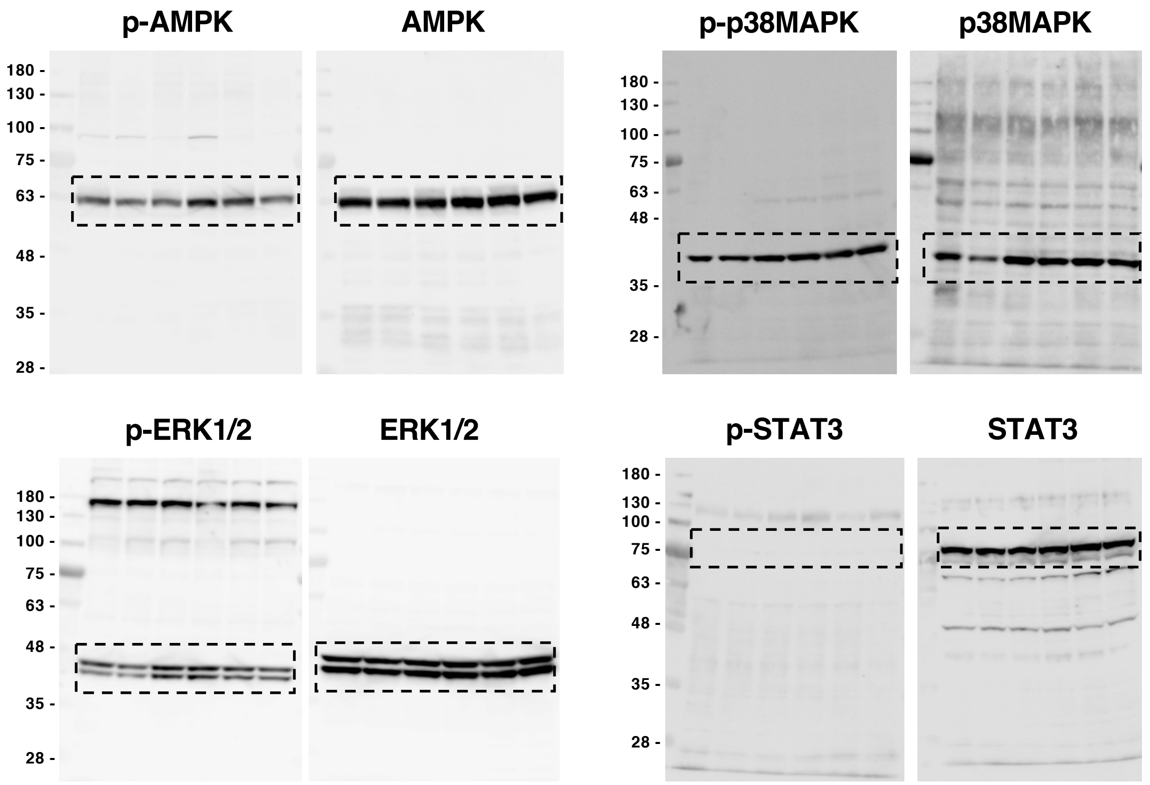

Fig. 5B

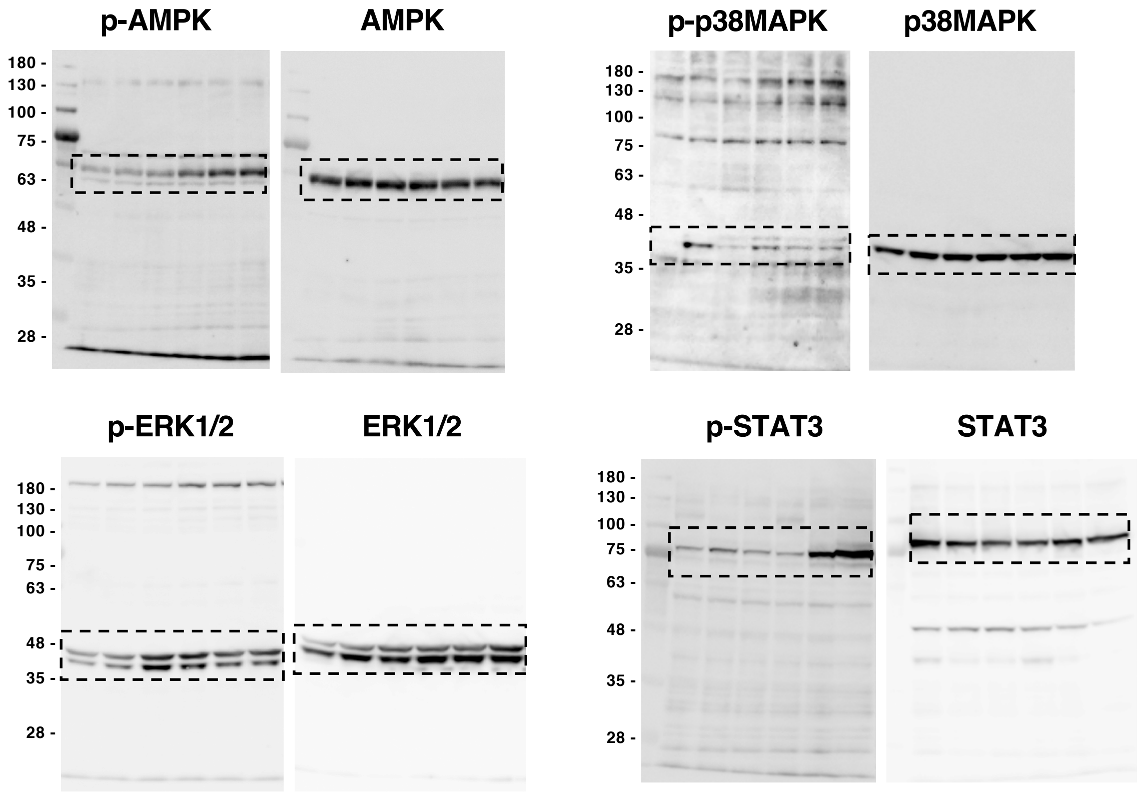

Supplementary Fig. S11-1. Uncropped gels for each Western blot.

Fig. 5F

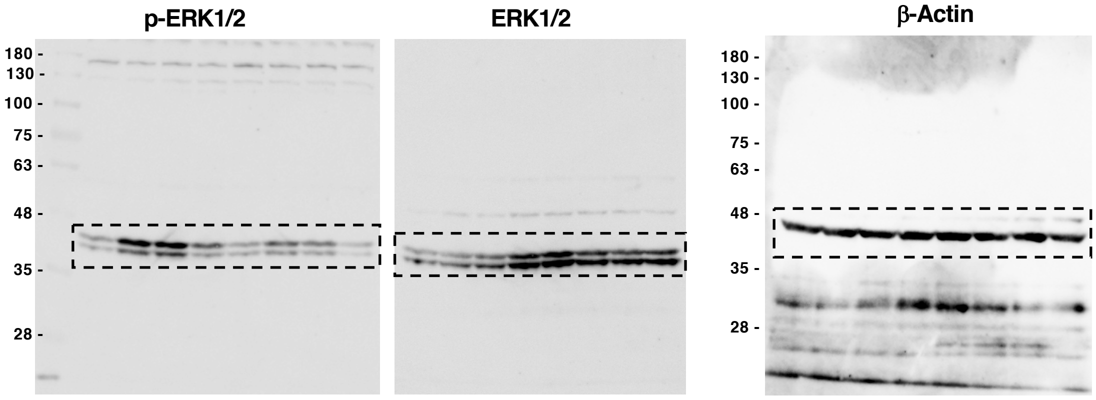

Supplementary Figure S11-2. Uncropped gels for each Western blot.
